# Supplementary material for: Enterovirus D68 capsid formation and stability requires acidic compartments
Source: mBio. 2023 Oct 11;14(5):e02141-23. doi: 10.1128/mbio.02141-23 (PMC10653823; doi:10.1128/mbio.02141-23)
Supplement: Supplemental Material — Figures S1 to S5. [file mbio.02141-23-s0001.pdf]

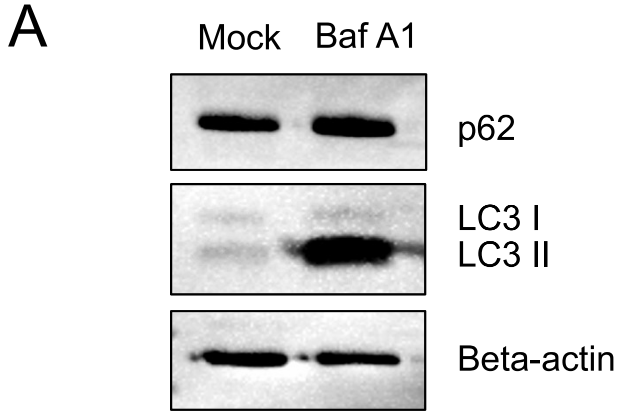

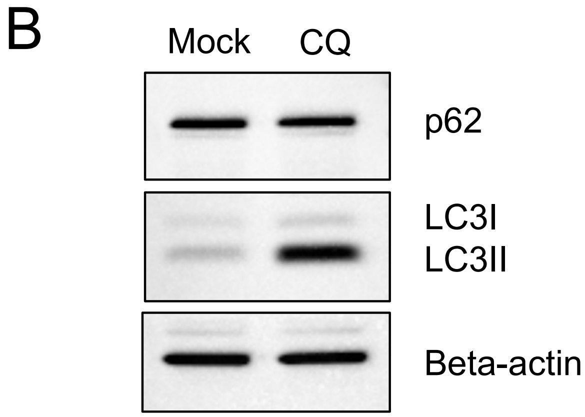

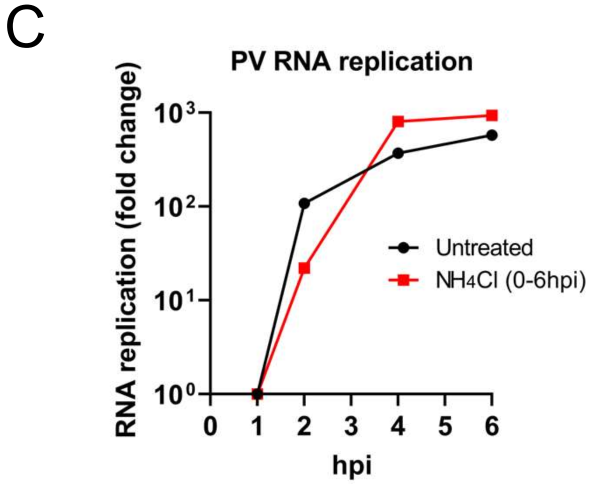

Suppl. Figure 1

Figure S1. Related to Figure 2. Effect of the acidification inhibitors on autophagy markers

Autophagy markers SQSTM1 and LC3B in mock samples of H1HeLas, untreated or treated with chloroquine (CQ; 100uM) (A) or Bafilomycin (B) for 6h, were analyzed by Western blot. Beta-actin served as a loading control. The gradual accumulation of LC3B served as an indicator of blocked autophagy.

(C) Effect of ammonium chloride treatment on PV RNA synthesis assessed by qRT-PCR. Cells were infected with PV at an MOI of 20 for 30 min at 37°C, then the residual virus was washed away, followed by either adding normal media (untreated infection, PV) or media containing 20mM of ammonium chloride (PV+NH_4Cl). Cells were then collected at the indicated time points after infection, and total RNA was extracted and subjected to qRT-PCR RNA analysis following cDNA synthesis. The level of GAPDH mRNA was used as an internal control.

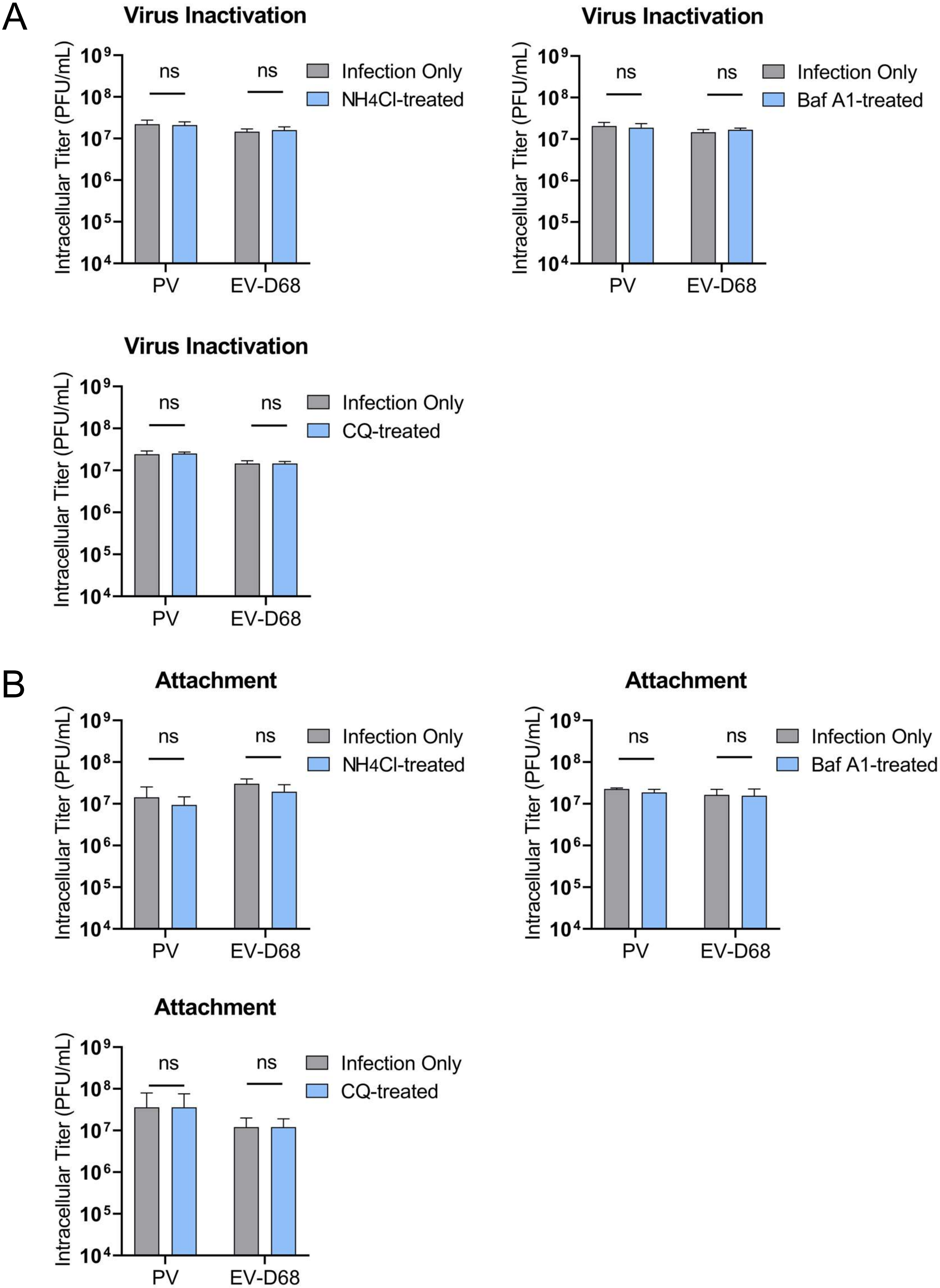

Suppl. Figure 2

Figure S2. Related to Figure 3. Neutralization and attachment assays using acidification inhibitors

(A) Effect of acidification inhibitors on virus particle neutralization. Viruses and each drug were incubated for 1h at 37°C. Dilution of 20x of each drug was performed to subtherapeutic concentration to prevent drug-related alteration of cellular functions. (B) Effect of acidification inhibitors on viral attachment. H1HeLa were pre-chilled before the infection of PV or EV-D68 at MOI 0.1. The virus binding/attachment step was allowed to run for 1h at 4°C. Then the residual virus was washed at least twice and the cells were moved at 37°C for 6 hpi.

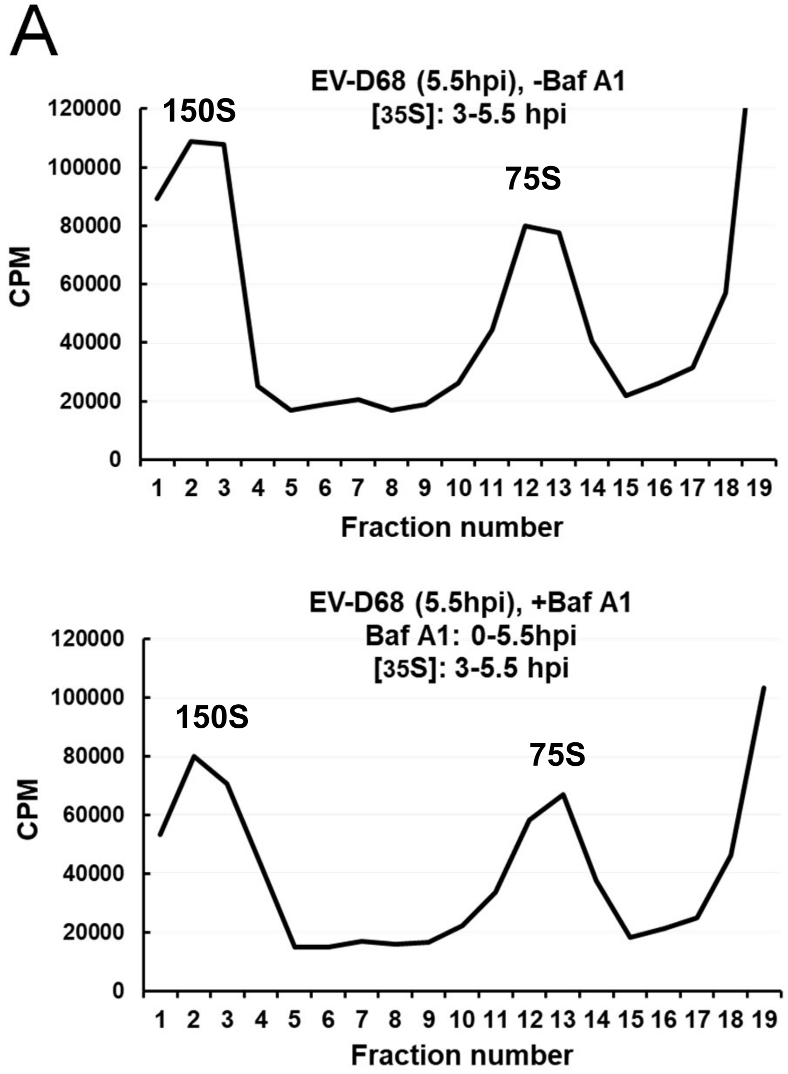

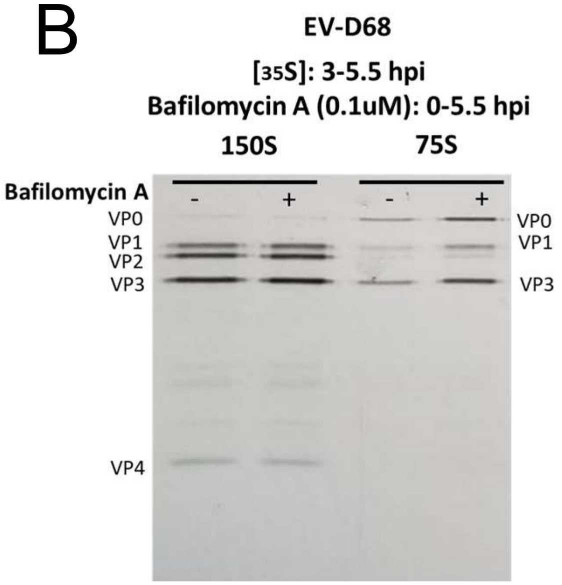

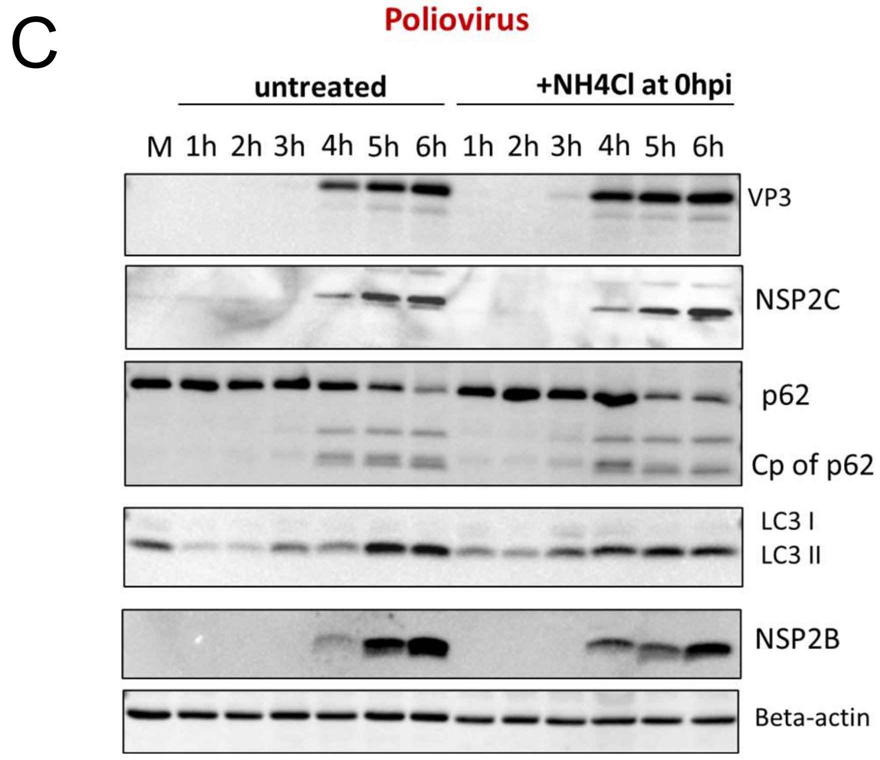

Suppl. Figure 3

Figure S3. Related to Figure 4. EV-D68 capsid production and virion maturation is not altered by treatment with Bafilomycin A

(A) H1HeLa were infected at an MOI of 20, and the half of samples were treated with Bafilomycin A (0.1uM) added at 0 hpi and continued until the end of infection (5.5 hpi). Cells were labeled with 35S-Methionine either from 3 hpi until collection at 5.5 hpi (A). Cellular lysates were separated on 15-30% freshly prepared sucrose gradients and subjected to ultracentrifugation. Fractions were collected using the Fraction System and the counts per minute (CPM) were measured for each fraction. The experiments were independently repeated three times and the representative gradients are shown.

(B) The collective three fractions of each determined peak (150S and 75S) were pooled and run on SDS-PAGE. The 35S-Methionine labeled bands were visualized using autoradiography films. The bands are labeled according to the expected relative migration pattern, while VP2 is identified by its absence in the 75S peak.

(C) Production of several non-structural proteins of PV is not affected by treatment with acidification inhibitors. H1HeLa cells were either untreated/mock (M) or infected with PV at an MOI of 20 for 6 h. At 0 hpi, the infected cells were washed, followed by either adding normal DMEM (PV -NH_4Cl) or DMEM with 20mM of ammonium chloride (PV; +NH_4Cl), and samples were collected every hour during infection. Samples were subjected to western blot analysis for traditional autophagy markers: LC3B, p62(SQSTM1), and its Cp (cleavage product) and viral proteins: VP3 (virus structural capsid protein 3), as well as non-structural proteins, such as 2C (NSP2C) and 2B (NSP2B). Beta-actin served as a loading control.

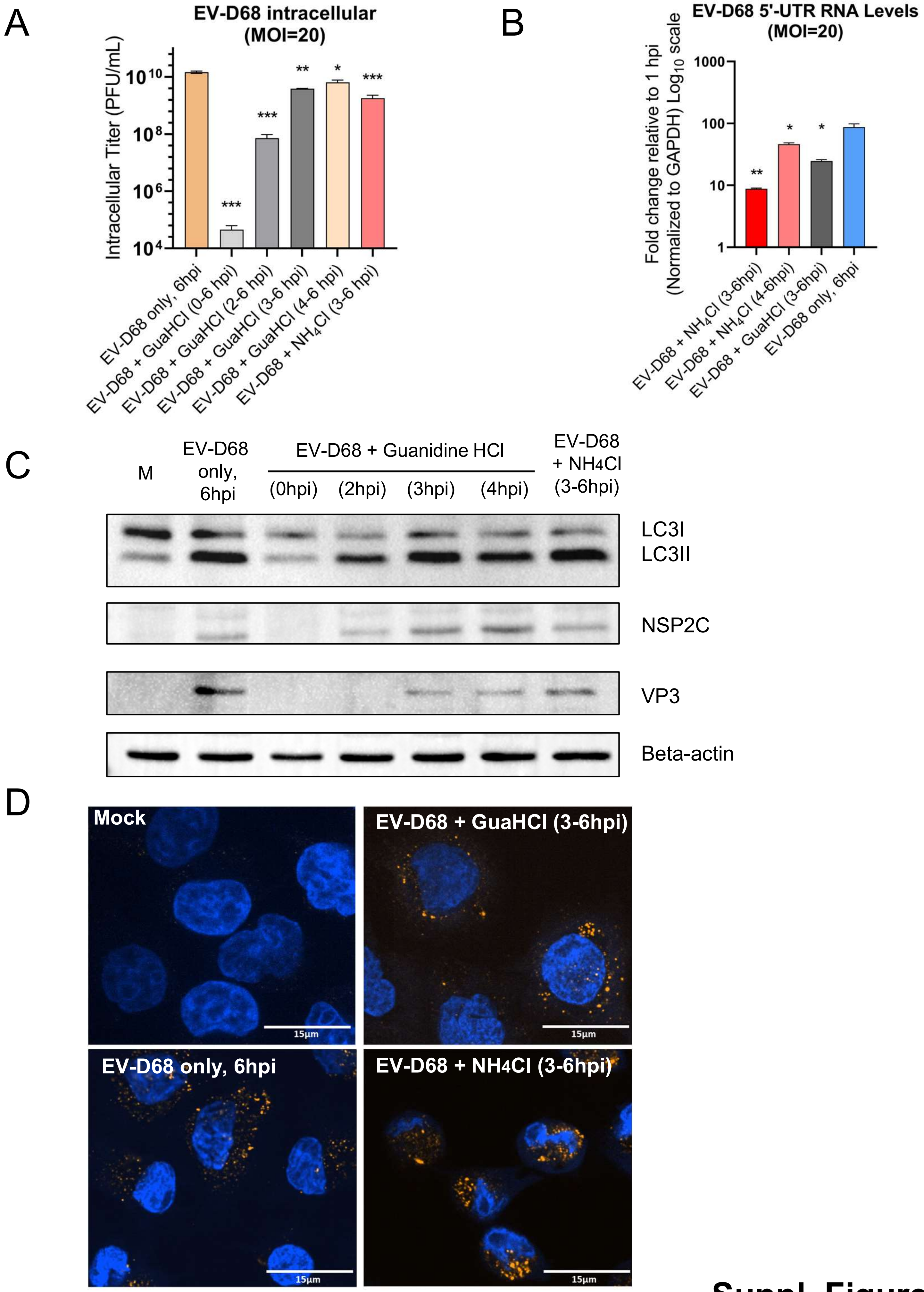

Suppl. Figure 4

Figure S4. Effect of ammonium chloride and guanidine chloride treatments on EV-D68 titer, RNA levels, and protein synthesis. Related to Figure 5.

(A) Titers of intracellular EV-D68 upon treatment with ammonium chloride (NH_4Cl, 20 mM) or guanidine chloride (GuHCl, 2mM) at indicated time points. H1HeLa were infected with EV-D68 at an MOI of 20; after 30 min of virus adsorption at 37°C (0 hpi), cells were washed twice to remove the initial virus input. At the indicated time points post-infection, ammonium chloride (20mM in complete DMEM) or GuHCl (2mM) was added to the cells until the end of the infection (6hpi). Control samples (EV-D68 only) were left untreated until virus collection at 6hpi. Intracellular titers were analyzed by plaque assay. Unpaired student's t-test was used for the statistical analysis (***= $p< 0.001$; **= $p< 0.01$; *= $p \leq 0.05$; ns=not significant).

(B) Effect of ammonium chloride (NH4Cl, 20 mM) and guanidine chloride (GuHCl, 2mM) on EV-D68 RNA levels assessed by qRT-PCR. Cells were infected with EV-D68 at an MOI of 20 for 30 min at 37°C, then the residual virus was washed away, followed by either adding normal media (EV-D68 only), or complete media containing 2 mM of guanidine chloride (EV-D68 + GuHCl), or 20mM of ammonium chloride at indicated time point until virus collection (6hpi). Untreated infection (EV-D68, 6hpi) served as a control. Total RNA was extracted from all samples and subjected to qRT-PCR RNA analysis following cDNA synthesis. Data represents relative expression of 5′UTR where samples are compared to the 1 hpi. All samples are normalized to GAPDH and have been log10 transformed. Unpaired student's t-test was used for the statistical analysis (***= $p< 0.001$; **= $p< 0.01$; *= $p \leq 0.05$; ns=not significant).

(C) Effect of the ammonium chloride treatment on EV-D68 protein synthesis. H1HeLa cells were either untreated/mock (M) or infected with EV-D68 at an MOI of 20 for 6 h. At 0 hpi, the infected cells were washed, followed by adding normal DMEM (EV-D68 only). At indicated time points, complete DMEM with 20mM of ammonium chloride (EV-D68 + NH_4Cl) or 2mM of guanidine chloride (EV-D68 + GuHCl), and samples were collected at 0, 2, 3, 4 and 6 hpi. Samples were subjected to western blot analysis for autophagy marker LC3B and viral proteins: VP3 (virus structural capsid protein 3), as well as non-structural protein 2C (NSP2C). Beta-actin served as a loading control.

(D) Confocal imaging of EV-D68 infection upon ammonium chloride or guanidine chloride treatment added during the transition point. H1HeLa were infected with EV-D68 (MOI=20) for 6h or left uninfected (mock). Infected cells were then left untreated (EV-D68 only), treated with ammonium chloride (EV-D68 +NH_4Cl, 3-6hpi) or guanidine chloride between 3 to 6hpi (EV-D68 + GuHCl, 3-6hpi). Cells were fixed and then stained for dsRNA (yellow) and nuclei (blue). Scale bar: 15 μM.

A

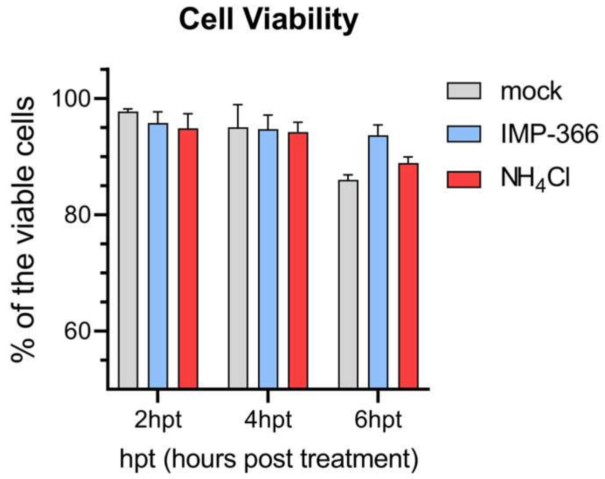

B

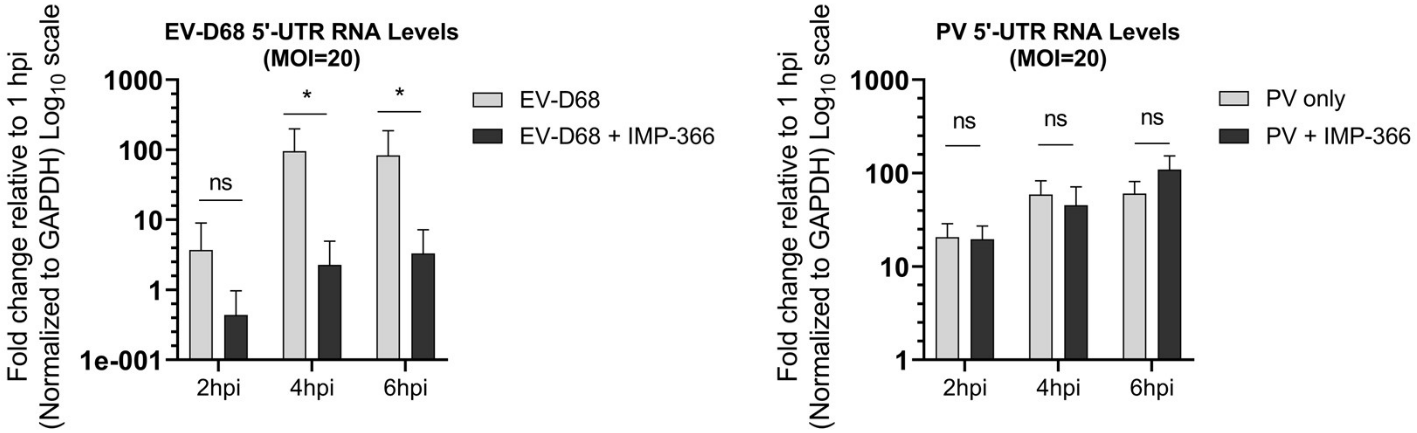

Suppl. Figure 5

Figure S5. Effects of continuous treatments on H1HeLa cell viability and EV-D68 and PV RNA levels during infection. Related to Figure 6.

(A) IMP-366 (5µM) and NH_4Cl (20mM) on H1HeLa cell viability (via trypan blue assay) at the indicated times. Each data point represents the mean ± SD, n = 3. The difference in viability among treated vs. untreated cells is not significant.

(B) Effect of IMP-366 on EV-D68 and PV RNA levels assessed by qRT-PCR. Cells were infected with EV-D68 or PV at an MOI of 20 for 30 min at 37°C, then the residual virus was washed away, followed by either adding normal media (EV-D68/PV only) or media containing 5µM of IMP-366 (EV-D68/PV; +IMP-366) at 0 hpi until virus collection (6hpi). Untreated infection (EV-D68/PV) served as a control. At indicated time points, cells were collected, total RNA was extracted from all samples and subjected to qRT-PCR RNA analysis following cDNA synthesis. Data represents relative expression of 5′UTR where samples are compared to the 1 hpi. All samples are normalized to GAPDH and have been log10 transformed. Unpaired student's t-test was used for the statistical analysis (***= p< 0.001; **= p< 0.01; *= p ≤ 0.05; ns=not significant).
